# Supplementary figures and images for: Multi-Omics Analysis of the Tumor Microenvironment in Liver Metastasis of Colorectal Cancer Identified FJX1 as a Novel Biomarker
Source: Front Genet. 2022 Jul 19;13:960954. doi: 10.3389/fgene.2022.960954 (PMC9343787; doi:10.3389/fgene.2022.960954)

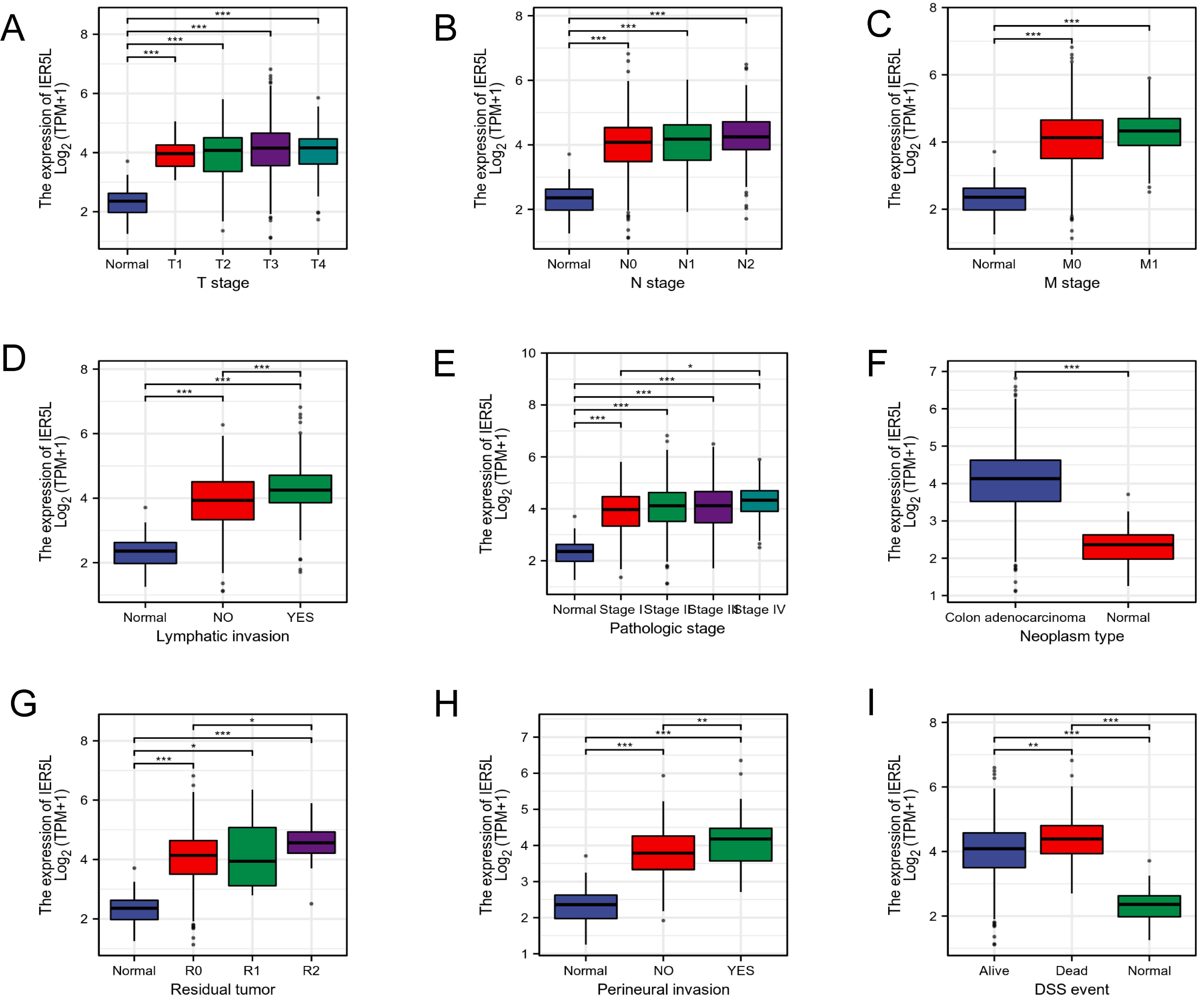

Supplement: Supplementary file 1 [file Image3.JPEG]

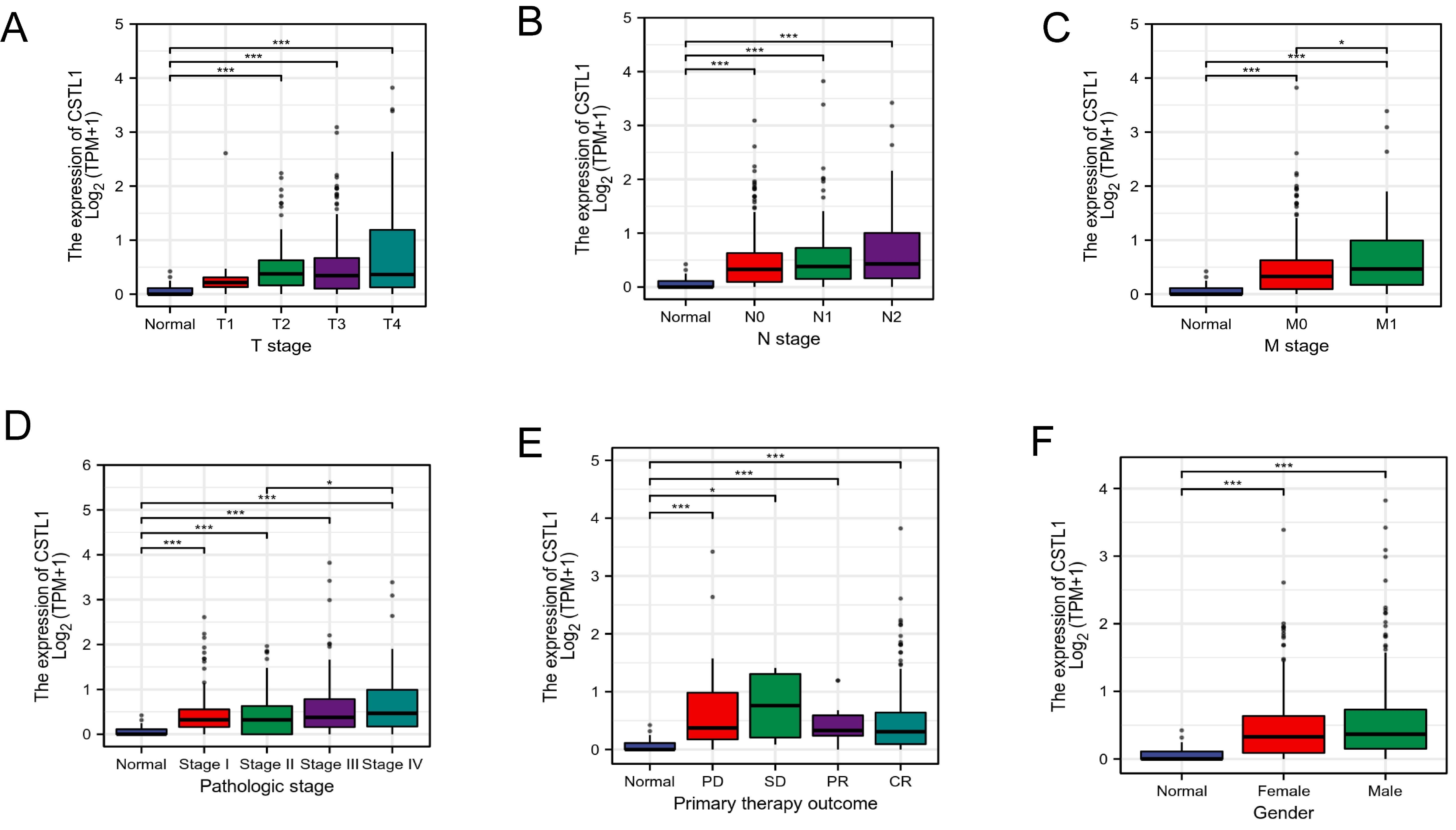

Supplement: Supplementary file 2 [file Image1.JPEG]

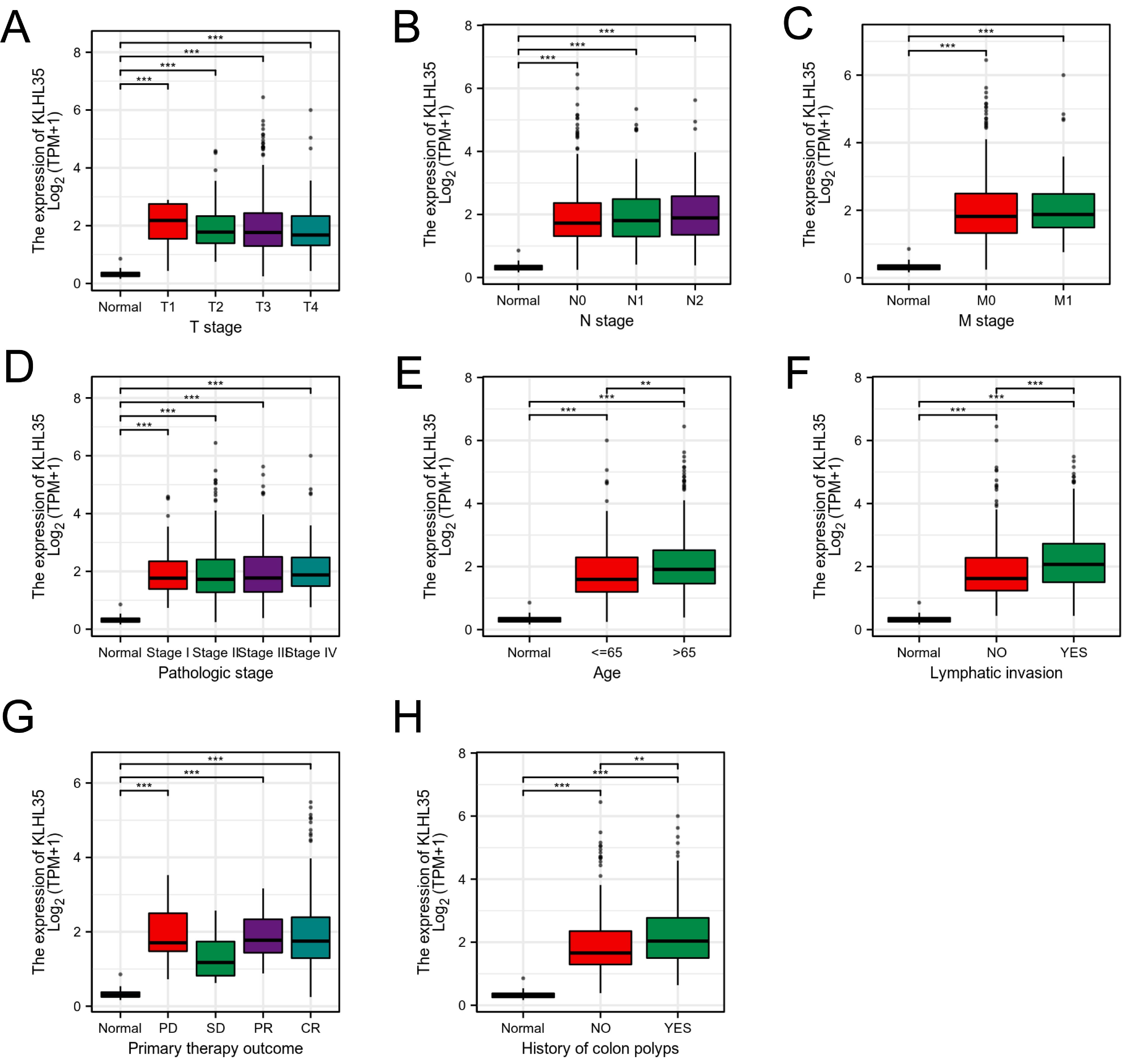

Supplement: Supplementary file 3 [file Image4.JPEG]

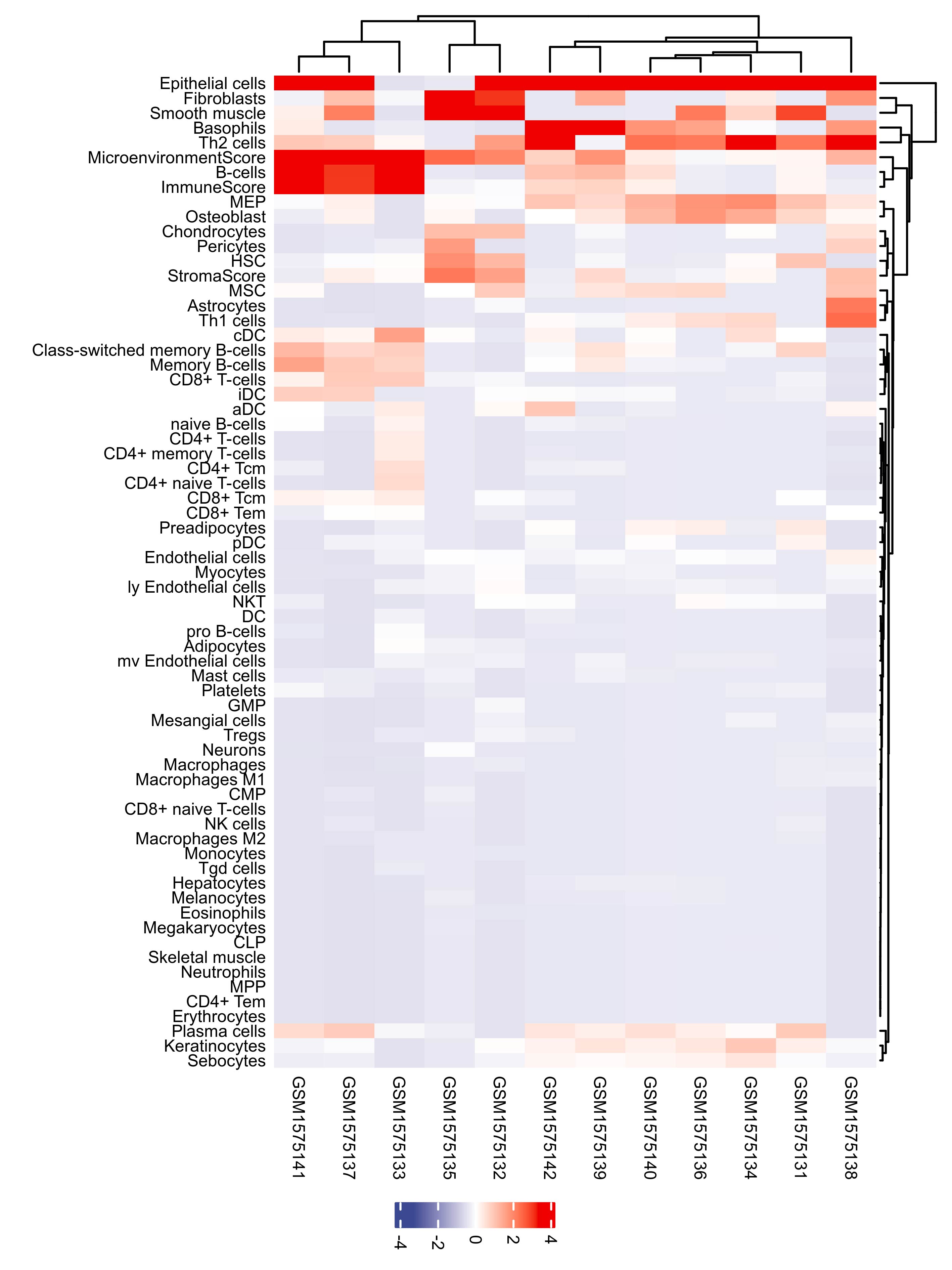

Supplement: Supplementary file 4 [file Image7.JPEG]

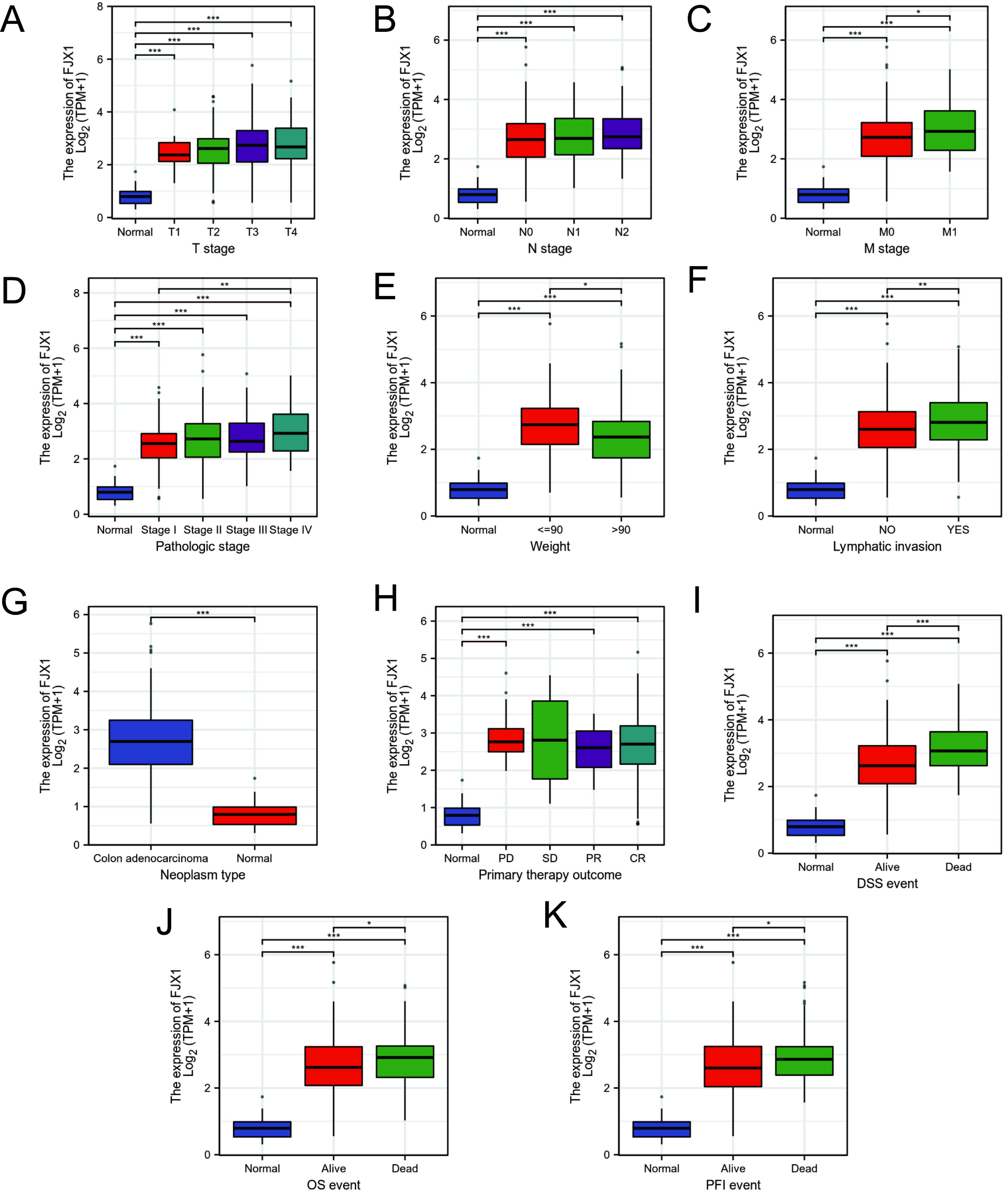

Supplement: Supplementary file 5 [file Image2.JPEG]
